# Changes in Modes of Social Contact and Their Links With Mental Health During the COVID-19 Pandemic

**DOI:** 10.1093/geroni/igab046.2061

**Published:** 2021-12-17

**Authors:** Laura Finch, Louise Hawkley

**Affiliations:** 1 NORC at The University of Chicago, Chicago, Illinois, United States; 2 NORC at the University of Chicago, Chicago, Illinois, United States

## Abstract

Amid the COVID-19 pandemic, social distancing has been emphasized for older adults because of their greater physical health risks. Using data from the National Social Life, Health and Aging Project (NSHAP), we examined how older adults may have changed their frequency of contact with others via various modes (i.e., in-person, phone calls, messages, and video calls) since the pandemic started, and how these choices may be impacting their mental health. From September 2020 through January 2021, NSHAP respondents (N=2,554 age 50-94 with data from 2015-16) completed a survey via web, phone, or paper-and-pencil. Although some older adults reported reducing their in-person contact with out-of-household family (38%) and friends (40%) since the pandemic started, some also increased contact with them via remote modes such as phone calls (25% and 16% with family and friends respectively); emails, texts, or social media messages (26 and 21%); and video calls (24 and 18%). Net of demographics, living alone, survey mode, and 2015-16 levels of the respective mental health variables, those who decreased in-person contact with family were less happy (B=-0.12, SE=0.06, p=.035), had higher loneliness scores (B=0.23, SE=0.09, p=.011), and more frequently felt depressed (B=0.10, SE=0.05, p=.055). In the presence of decreased in-person contact, increases in remote modes of contact had no net remediating impact—a pattern also found when analyzing contact with friends. Results indicate a persistent adverse effect of reduced in-person contact on mental health despite increased contact with family and friends via remote means.

